# Progestins as Anticancer Drugs and Chemosensitizers, New Targets and Applications

**DOI:** 10.3390/pharmaceutics13101616

**Published:** 2021-10-04

**Authors:** Tatiana A. Fedotcheva, Nadezhda I. Fedotcheva, Nikolai L. Shimanovsky

**Affiliations:** 1 Science Research Laboratory of Molecular Pharmacology, Medical Biological Faculty, Pirogov Russian National Research Medical University, Ministry of Health of the Russian Federation, Ostrovityanova St. 1, 117997 Moscow, Russia; shimannn@yandex.ru; 2 Institute of Theoretical and Experimental Biophysics, Russian Academy of Sciences, Institutskaya str., 3, Pushchino, 142290 Moscow, Russia; nfedotcheva@mail.ru

**Keywords:** progesterone P_4_, progestins, cancer, P-glycoprotein, multidrug resistance, mitochondrial permeability transition pore, Wnt/β-catenin

## Abstract

Progesterone and its synthetic analogues, progestins, participate in the regulation of cell differentiation, proliferation and cell cycle progression. Progestins are usually applied for contraception, maintenance of pregnancy, and hormone replacement therapy. Recently, their effectiveness in the treatment of hormone-sensitive tumors was revealed. According to current data, the anticancer activity of progestins is mainly mediated by their cytotoxic and chemosensitizing influence on different cancer cells. In connection with the detection of previously unknown targets of the progestin action, which include the membrane-associated progesterone receptor (PR), non-specific transporters related to the multidrug resistance (MDR) and mitochondrial permeability transition pore (MPTP), and checkpoints of different signaling pathways, new aspects of their application have emerged. It is likely that the favorable influence of progestins is predominantly associated with the modulation of expression and activity of MDR-related proteins, the inhibition of survival signaling pathways, especially TGF-β and Wnt/β-catenin pathways, which activate the proliferation and promote MDR in cancer cells, and the facilitation of mitochondrial-dependent apoptosis. Biological effects of progestins are mediated by the inhibition of these signaling pathways, as well as the direct interaction with the nucleotide-binding domain of ABC-transporters and mitochondrial adenylate translocase as an MPTP component. In these ways, progestins can restore the proliferative balance, the ability for apoptosis, and chemosensitivity to drugs, which is especially important for hormone-dependent tumors associated with estrogen stress, epithelial-to-mesenchymal transition, and drug resistance.

## 1. Introduction

Progestins (gestagens) are synthetic sex steroid hormones, analogues of progesterone. For decades, progestins have been traditionally used for contraception, maintenance of pregnancy with threatened miscarriage, in hormone replacement therapy, and assisted reproductive technology procedures. Recently, a number of studies have also revealed their effectiveness in the treatment of endometriosis and hormone-sensitive tumors [1,2,3].

There is substantial evidence indicating that progestins regulate the proliferation and invasion of epithelial ovarian and endometrial cancer cells [1,4]. The efficiency of progestins, in particular the pregnane progestins medroxyprogesterone acetate (MPA) and megestrole acetate (MA), in the treatment of endometrial and cervical cancers is well established [5]. With regard to the use of progestins in breast cancer, the problem is still under investigation, since progestin therapy can have controversial results in relation to triple-negative (progesterone receptor (PR), estrogen receptor (ER) and HER2 receptor-negative) and receptor-positive breast cancer status. In connection with the detection of previously unknown targets of progestin action, which include membrane-associated PR, mitochondrial PR, membrane transport proteins, the mitochondrial permeability transition pore (MPTP) and checkpoints of signaling pathways, new aspects of the clinical use of progesterone and its synthetic analogues have emerged.

The actively developing direction in the creation of a new class of antitumor agents aimed at new targets such as P-glycoprotein (P-gp) and MPTP has led to the appearance of principally new drugs. Recently, the P-gp inhibitor Tariquidar^®^ and the Bcl-2 protein inhibitor Venetoclax^®^ have been approved by the FDA and introduced into clinical practice [6,7]. At the stage of preclinical studies are TSPO-inhibitors, blockers of cyclophilin D, and other components that form MPTP [8]. The steroid structure and unique action on proliferation and apoptosis provide some evidence that progestins can also affect these targets. However, the cytostatic and chemosensitizing action of gestagens on different types of cancers, especially hormone-dependent tumors, and their ability to increase the anticancer activity of the classical cytostatics doxorubicin and cisplatin have not yet been clinically studied.

Table 1 lists some pregnane progestins which exhibit anticancer and chemosensitizing activities in vivo. These include pregnane-based compounds with various substituents at the C3 and C17 carbon atoms of the steroid. According to the current classification, considering the structural features, this group belongs to pregnane progestins derived from progesterone [9]. These progestins act predominantly on PR. The affinity of some of them to PR and their specific progestogenic activity can even exceed that of progesterone [10,11]. As a rule, they retain specific activity for a longer period then progesterone and do not exhibit androgenic and estrogenic activities [12]. The group of pregnane progestins includes newly synthesized 17a-acetoxy-3b-butanoyloxy-6-methyl-pregna-4,6-diene-20-on (gestabutanoyl, butagest) and other modified in C3 position progesterone analogues, which reveal anticancer activities in experimental tests [5]. Two other groups of progestins, estranes and gonanes derivatives, demonstrate the androgenic activity and low affinity for the receptor [12]. These derivatives and other widely used progestins, such as dydrogesterone (pregnane, a *stereoisomer* of *progesterone)* and dienogest (ethylgonane), having a low affinity for PR and low gestagenic activity, are actively used in gynecological practice [9]. Moreover, according to their chemical structure, various progestogens may bind to other members of the nuclear receptor superfamily, e.g., androgen receptors, glucocorticoid receptors, and mineralocorticoid receptors, and may act as agonists or antagonists [11].

Besides the progestogenic action, pregnane derivatives have specific effects [9,10]. These additional properties can underlie their possible application in the treatment of hormone-dependent tumors. In the course of studies of progestins as antineoplastic agents, their new important targets associated with the processes of proliferation and apoptosis have been identified.

In this review, we summarized the data on the cytostatic and chemosensitizing effects of different progestins and possible mechanisms of their action, including our data obtained with the newly synthesized pregnane progestin gestobutanoyl, a derivative of 17-acetatemepregenol. It is assumed that the cytostatic and chemosensitizing effects of progestins is predominantly associated with: (1) their own cytotoxic effect on some hormone-dependent tumors (breast cancer, endometrial cancer, cervical cancer); (2) modulation of the expression and activity of multidrug resistance (MDR)–related proteins; and (3) the inhibition of survival signaling pathways that activate the proliferation and MDR protein up-regulation in cancer cells.

## 2. Gestagens as Cytotoxic Drugs, Possible Mechanisms and Targets

In the last decades, among all approved progestins, synthetic derivatives of P_4,_ megestrol acetate (MA) and medroxyprogesterone acetate (MPA) have been recommended for use in the treatment of different types of cancers. Their effectiveness was confirmed in clinical trials in regard to a complete response rate and pathological remission (Table 2). In PR-transfected or estrogen receptor-negative breast cancer cells, DNA synthesis and cell proliferation are markedly inhibited by P_4_ treatment, indicating a receptor-dependent role for P_4_ in tumor growth inhibition [13]. MA and MPA have been successfully used to treat advanced breast cancer, endometrial cancer, prostate cancer, and endometrial hyperplasia and are approved as anticancer drugs [14].

The mechanisms of antitumor activity of progestins are still poorly understood, since their effects are often opposite and depend on the type of tissue and individual status of the expression of PR, ER, HER as well as WNT receptors. How progestins affect proliferation, estrogen signaling and WNT signaling are still being investigated (Table 2).

In clinical studies, both monotherapy with progestins and their combinations with other antitumor agents have been examined. The results of some trials indicated the effectiveness of cancer therapy with the application of progestins [15,16,17,18].

Based on the available data, three main directions of the antitumor activity of progesterone and its analogues can be distinguished. They are associated with the regulation of ER expression, modulation of proliferative cell signaling, and triggering of mitochondrial-dependent apoptosis.

### 2.1. Inhibition of ER Expression

**Figure 1:**
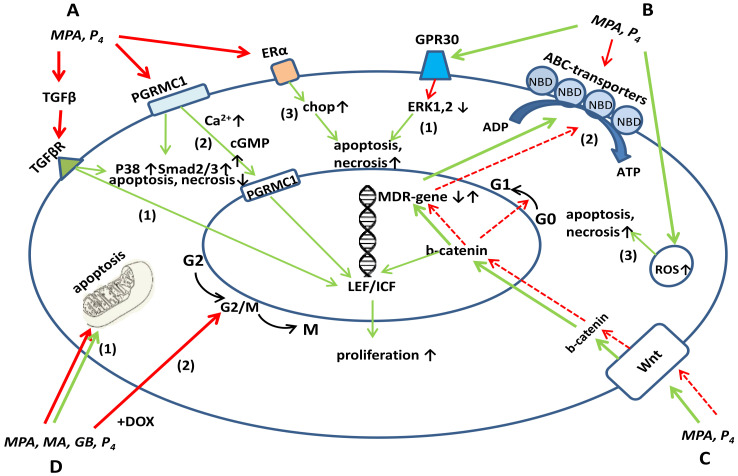
Influence of progestins on signaling pathways leading to the proliferation, apoptosis and drug resistance. (**A**). Progesterone and progestins decrease the TGF-β production and expression of TGF-β-target genes SMAD, P38, NFkB, RAS, PI3K (1), decrease PGRMC1 expression, which attenuates the Ca^2+^- and cGMP-mediated proliferative signals to the nucleus and the PGRMC1-mediated resistance to drugs (2), cause ER down-regulation, decrease estrogen-linked mitosis, and increase CHOP and FOXO1 expression, thereby inducing apoptosis and necrosis (3). (**B**). Progestins promote apoptosis and necrosis by down-regulating ERK-1,2 through GPR30 (1); inhibit the expression and transport activity of MDR-related proteins P-gp, BCRP, MRP through the interaction with the nucleotide-binding domain (NBD) (2); cause ROS formation and apoptosis (3); (**C**). Progestins at low concentrations promote (solid line) and at high concentrations (dashed line) inhibit Wnt signaling linked through the MDR-promoter element with MDR. (**D**). Progestins suppress cell cycle progression at the G_o_G_1_ checkpoint and in combination with cytostatics (DOX) induce G2/M block (1), regulate the MPTP opening and mitochondrial-dependent apoptosis (2). Green line-activation, red line-inhibition of indicated processes.

It is known that estradiol, due to its receptors, regulates the proliferation of target cells [19]. Therefore, the imbalance between the expression of PR and ER is of key importance in tumor transformation. Regulation of the expression of PR and ER by progestins is one of the mechanisms of their cytostatic action (Figure 1A).

As was shown, progestins decrease the number of progesterone receptors nPR-A and estrogen receptors ER-β [19]. A model of the impact of the normal and impaired hormonal cycle on cervical cancer is proposed. It is known that ERα and ERβ intensify proliferative activity of cancer and non-cancer cells via estradiol binding [20]. The stimulating effect of E_2_ is mainly promoted through stromal ERα. Presumably, epithelial PRs mediate, at least partially, the tumor-suppressive function of P_4_, and hence high P_4_ has little effect on PR-negative cancer cells [21]. The results in the cervical cancer mouse model system suggest that if the cancer stroma expresses both ERα and PR, co-treatment with SERMs and selective PR modulators such as MPA may be synergistic [21].

Recently, the transition from endoplasmic reticulum stress to apoptosis and its regulation by progestins was revealed. It was shown that MPA induces CHOP expression [22]. CHOP is the best-characterized factor in the transition from endoplasmic reticulum stress to apoptosis. CHOP is expressed at low levels under physiological conditions, but strongly increases under severe and prolonged endoplasmic reticulum stress. In PR isoform B-positive Ishikawa cells treated with MPA, endoplasmic reticulum stress-related CHOP and HERPUD1 proteins were both highly expressed, which indicates their participation in apoptosis caused by MPA [22]. In this case, MPA apparently activates endoplasmic reticulum stress by the PR isoform B pathway.

### 2.2. Inhibition of Epithelial-to-Mesenchymal Transition (EMT)

In EMT, cells acquire migratory and invasive properties inherent in rapidly proliferating low-differentiated stem cells. Simultaneously, the processes of apoptosis are inhibited and immunosuppression occurs [23]. P_4_ is known to inhibit EMT [24]. Central to EMT is the activation of important signaling pathways such as Wnt/β-catenin and TGF-β. Progesterone inhibits EMT and metastatic spread of endometrial cancer by stimulating T-cell infiltration. The progression of disease is characterized by both loss of progesterone signaling and T-lymphocytes driven immunosuppression, as well as the modulation of pathways reminiscent of EMT and the transition from the epithelial phenotype to a more invasive mesenchymal phenotype. P_4_ inhibits EMT partly owing to its inhibitory effect on TGF-β. This process is very important, particularly in the case of endometrial cancer. It was shown also that progesterone in vitro inhibits TGF-β signaling 72 h after treatment of Ishikawa endometrial cancer cells and effectively suppresses the viability and invasion of endometrial cancer cells with increased E-cadherin expression [25]. Thus, progesterone inhibits EMT and stimulates immune protection, increasing the production of tumor-infiltrating lymphocytes [24].

### 2.3. Inhibition of PI3K/AKT, Ras/Raf/MEK/ERK, WNT/β-Catenin Cell Signaling Pathways

Based on current data, it can be assumed that P_4_ regulates the most ancient signaling pathways of proliferation and apoptosis, including PI3K/AKT, Ras/Raf/MEK/ERK, WNT/β-catenin (Figure 1A). Presuppositions for this specific regulation follows from the evidence that progesterone regulates the menstrual cycle, causing endometrial atrophy and exfoliation during the luteal phase of the cycle, and the termination of excessive proliferation of the endometrial tissue during pregnancy [26].

The influence of progestins on the signaling pathways of proliferation follows in particular from the fact that they do not induce instant cell death, but only throughout a certain period after a treatment. It can be assumed that the inhibition of cell growth in the presence of progestins is due to a slowdown in replication processes [27]. This is indicated by the data showing that the cytostatic effect of progestins develops on the 4th–6th day of the incubation with cells of different cancer lines. Unlike progestins, doxorubicin at a concentration of 50 µM causes the inhibition of the growth of MCF-7/WT cells by more than 50% already after 48 h of incubation. Thus, for the cytostatic effect of doxorubicin to occur, about two cell cycles must pass [27]. Most likely, the cytostatic effect of progestins is not associated with a rapid damaging effect on the DNA of the tumor cell, since otherwise the percentage of dead cells would be high already after 48 h of incubation, as is the case with classical cytostatics. Besides, the synthetic progestin MPA has a biphasic effect on the human breast cancer T-47D line, stimulating the proliferation after 24–48 h of incubation and inhibiting it at 72 h of incubation [28]. Progesterone at concentrations from 1 µM to 100 µM caused nuclear fragmentation, which depended on both the duration of cultivation and the concentration in nPR-positive breast cancer (MCF-7), and in nPR-negative cervical cancer (C4-I) [29].

One of the progestins’ targets in tumor cells is the so-called G protein-coupled receptor (GPR)30, a receptor associated with G-protein 30 [30]. It is proposed that the up-regulation of this receptor by the progestin MPA leads to the inactivation of ERK-1 and ERK-2; as a result, MPA induces cell death (Figure 1B). ERK-1 and ERK-2 are mitogen-activated protein (MAP) kinases, key enzymes that regulate signaling cascades of reactions involved in the processes of cell proliferation and death [30]. ERK-1 and ERK-2 are most characteristic of breast cancer cells [31]. It is important to note that a decrease in the activity of ERK-1 and -2 is already observed 24 h after the injection of MPA; therefore, the cell death recorded by the MTT and ^(3)^H-thymidine tests only on days 4-6 of incubation is a result of not only changes in the activity of kinases, but also of other cellular processes regulated by progestins [27].

The PI3K/AKT pathway and Wnt/β-catenin pathway are the ancient pathways which activate proliferation [32], and they can both be inhibited by P_4._. It was shown that in the process of decidualization of human endometrial stromal cells, a downregulation of Akt isoforms as well as a decrease of Akt activity occurs (Figure 1A,C). This fact also demonstratesPI3K/AKT pathway inhibition by high dose progesterone since decidualization is associated with the transition of the endometrium from the proliferative phase to the secretory phase [33]. On the contrary, with low or no PR expression, progesterone activates the PI3K/AKT pathway and promotes resistance. Only in the presence of PR progesterone does inhibit the PI3K/AKT pathway and suppress proliferation, as was demonstrated on Ishikawa endometrial cancer cells [34]. Thus, with the availability of PR, the PI3K/Akt pathway and the proliferative balance are mildly regulated by progesterone.

The Wnt/β-catenin pathway plays a key role in the proliferation and differentiation of any type of cells, including stem cells. The inhibition of the Wnt/β-catenin signaling pathway has already become the subject of preclinical studies. Some of the compounds tested, such as the anti-helminthic drugs Niclosamide and Pyrvinium, as well as the non-steroidal anti-inflammatory drug Sulindac were approved by the FDA [35]. Some experimental data from preclinical studies have already shown positive effects, for example a decrease in chemoresistance to fluoropyrimidine and platinum compounds when using the Wnt inhibitor genistein, a soy isoflavone [36].

The direct inhibitory effect of P_4_ on Wnt/β-catenin signaling pathway via the activation of the progesterone receptors was shown on adrenocortical carcinoma (ACC) primary cultures and NCI-H295R cells [37]. Progesterone significantly inhibited the β-catenin migration into the nucleus. The functional effect of the β-catenin translocation is the down-regulation of the expression of some β-catenin target genes, namely MYC and survivin. These data indicate the involvement of β-catenin inhibition in the progesterone-induced apoptosis of ACC cells.

Further evidence comes from studies showing that progesterone is able to inhibit the Wnt/β-catenine pathway in endometrial carcinoma [38] (Figure 1C). The antitumor effect of progesterone is provided through its regulatory action on the so-called long non-coding RNA, NEAT1/microRNA-146b-5p, which mediates the WNT/β-catenin signaling pathway [38]. It was shown that incubation with 20 μM progesterone significantly decreased the expression level of the NEAT1, miR-146b-5p, LEF1, c-myc, and MMP9 genes of the WNT/β-catenin signaling pathway in Ishikawa endometrial cancer cells, wherein the cell cycle was inhibited in the G0/G1 phase [38]. The regulation of the Wnt signaling pathway was further confirmed by the data showing that progesterone induces the expression of FOXO1, a Wnt inhibitor [39]. FOXO1 has been shown to interact directly with the progesterone receptor to coordinate cell cycle regulation and differentiation of human endometrial stromal cells [40]. Furthermore, FOXO1 is also able to interact with β-catenin, thus directly inhibiting the Wnt/β-catenin signaling (Figure 1A). A moderate balance between estrogen and progesterone signaling underlies the proper functioning of the female reproductive tract and, in particular, the monthly re- and degenerative phases characteristic of the menstrual cycle. Wang Y. et al., proposed that the canonical Wnt/β-catenin signaling pathway may underlie this finely tuned hormonal equilibrium in endometrial homeostasis and, upon own constitutive activation, can lead to neoplastic transformation of the endometrium. During the menstrual cycle, estradiol enhances Wnt/β-catenin signaling in the proliferative phase, and during the secretory phase, progesterone inhibits Wnt/β-catenin signaling, thus restraining the proliferative action of estrogens. In the event of a loss of inhibitory progesterone signaling and increased estrogen signaling, persistent activation of Wnt/β-catenin signaling will trigger endometrial hyperplasia, which can develop into endometrial cancer [39].

Synthetic progestins, as potential Wnt inhibitors, need further research to determine their dosage and the mode of therapy, whether it be pulse or continuous. Since the activation of the Wnt/β-catenin signaling is one of the factors in the development of MDR, the inhibitory effect of progesterone on this signaling pathway may also be a promising aspect in the clinical use of progestins as chemosensitizers [41]. The cross-talk of the Wnt/β-catenin signaling pathway with MDR-responsive elements should lead to the expression of MDR-related genes and, conversely, the negative regulation of WNT/β-catenin signaling pathway should result in MDR suppression. Apparently, the regulation of proliferative signals by progesterone is a complex cascade process that is in some equilibrium with antiproliferative processes, and the equilibrium can shift towards proliferation or cell death, depending on the target tissue. Thus, the role of P_4_ and its receptors (PRs) in breast cancer etiology remains controversial. In breast cancer, progestins initiate a non-classical signaling of membrane progesterone receptors (mPRs) and progesterone receptor membrane component 1 (PGRMC1) by activating downstream targets, protein kinase c (PKC), protein kinase a (PKA), cyclic guanosine monophosphate (cGMP), and AKT, leading to Ca^2+^ influx, proliferation, and cell survival [42] (Figure 1A).

On the other hand, the membrane receptor PGRMC1 can block the proliferative cascade in breast cancer cells [43]. So, P_4_ (1 μM) inhibits the growth of PGRMC1 containing MDA-MB-231 cells. In PGRMC1-positive cells, P_4_ induces a stronger uptake of Ca^2+^ than in cells lacking PGRMC1. Therefore, the effect of P_4_ depends on the expression of membrane progesterone receptors and on Ca^2+^ fluxes, which also indicates a direct regulatory effect of P_4_ on signaling pathways. So, both genomic and non-genomic effects of progesterone can mediate the cytotoxicity [37] (Figure 1A). In another study, progesterone derivatives pregna-D’-pentaranes inhibited the proliferation of both PR-negative MDA-MB-453 cells and PR-positive MCF-7 cells, but the inhibitory effect was stronger in PR-positive MCF-7 cells [44].

### 2.4. Inhibition of TGF-β Production and Signaling

The inhibition of TGF-β production by high-dose progesterone is associated with phosphatidylinositol 3-kinase/protein kinase B (PI3K/AKT) signaling inhibition since this pathway is activated by TGF-β [45]. There is a direct link between TGF-β signaling pathway, apoptosis, and unique regulation of these processes by steroid hormones [46]. Progesterone can act as a regulator of alternative splicing of the TGF-β receptor gene [19]. P_4_ induces a switch from TGF-β 1 to TGF-β 2/3 expression; therefore, TGF-β 1 production begins to decrease [46]. Antitumorogenic effects of P_4_ have been confirmed by the induction of alternative expression of TGF-β isoforms in ovarian surface epithelium (OSE) [47]. When ovaries from the control and estrogen-only-treated monkeys were compared to the ovaries of progestin-treated monkeys (progestin levonorgestrel-treated), a marked decrease in the expression of TGF-β 1 and a concomitant increase in the expression of the TGF-β 2/3 isoforms was observed in the OSE. Apparently, heterodimers of TGF-β 2/3 isoforms do not trigger the activation of other signaling pathways as efficiently as homodimers. This alternative expression highly correlated with the increased apoptotic index in the OSE [47]. Also, a strong and direct relationship was demonstrated between P_4_ action and Fas/FasL signaling in normal and malignant OSE cells [47]. Relatively high doses of P_4_ (1 μM range) were used in this study to achieve the growth inhibition and apoptosis. Higher doses of P_4_, which can be achieved during pregnancy or by oral contraception, induced the cell cycle arrest or apoptosis in normal OSE cells and ovarian cancer (OCa) cells. Interestingly, P_4_ at low concentrations (10^−11^ to 10^−10^ M) was stimulatory to human OSE and OCa cell growth, but at high doses (10^−8^ to 10^−6^M), it exerted a marked inhibitory effect [47]. In all cases, co-treatment of a cell culture with P_4_ and its antagonist blocked the effect of the hormone, confirming the specificity of the hormonal action [47]. Taken together, these data support the hypothesis that reproductive states associated with rising levels of sex hormones promote cell proliferation in the normal OSE, which can contribute to the neoplastic transformation. Conversely, those states attended by high levels of circulating P_4_, as in the case of pregnancy, induce a loss of OSE cells and a protection against ovarian carcinogenesis [48].

### 2.5. Cell Cycle Inhibition in the G0/G1 Phase

Since P_4_ regulates cell signaling, it also should influence cell cycle progression (Figure 1D). The G0/G1 cell cycle arrest caused by progesterone is a consequence of the cell signaling regulation described above. When used alone, progesterone causes G0/G1 cell cycle arrest in endometrial cancer cells through regulating NEAT1 and miRNA-146b-5p [38]. NEAT1 has been reported to regulate the proliferation and mobility of several types of cancers, including colorectal cancer, pancreatic cancer, and hepatocellular carcinoma [49]. NEAT1 promotes the proliferation, migration, and metastasis of human breast-cancer cells by inhibiting miR-146b-5p expression. After treatment with progesterone, miR-146b-5p was notably upregulated in Ishikawa cells [38]. MiR-146b-5p is a direct target of NEAT1 and inhibits also the proliferation of breast cancer cells [49]. So, if P_4_ can stimulate miR-146b-5p expression in endometrial and breast cancer cells, it will down-regulate cell cycle progression. The role of miRs is post-transcriptional gene regulation via pairing with the 3′untranslated region (UTR) of target messenger RNAs (mRNAs), leading to mRNA degradation or translation blockage.

Another mechanism of P_4_ influence on the cell cycle is the suppression of the cell propagation and G0/G1 cell cycle progression through inhibition of LEF1 and downstream genes c-myc and MMP9 in the Wnt/β-catenin signaling pathway [38].

It was previously shown that progesterone and its analogues, in combination with cytostatic compounds, have a distinct influence on cell cycle progression. In these cases, the amount of non-surviving cells greatly increased in a super-additive manner. In combination with doxorubicin, they caused G2/M-arrest in MCF-7 cancer cells [50]. Also G2/M block has been demonstrated in the case of the combination of progesterone with alsterpaullone in endometrial cancer cells [51].

### 2.6. Regulation of Mitochondrial-Dependent Apoptosis

Progesterone and its synthetic analogues can trigger the mitochondrial pathway of apoptosis [52]. The MPTP opening is considered as a common pathway of cell death through the development of apoptosis, necrosis, and, possibly, ferroptosis [53]. A drop in the mitochondrial membrane potential leads to the formation of reactive oxygen species, the release of apoptogenic factors, and the activation of caspases, serving as a signal for the activation of the final effector stage of apoptosis. The existing models of the mitochondrial implementation of the apoptotic program take into account the activity of regulatory proteins of the family Bcl-2, adenine nucleotide carriers (ANT), and the voltage-dependent anion channel (VDAC) [54]. It has been established that steroid hormones, progesterone in particular, produce extragenomic effects on mitochondrial processes associated with apoptosis induction [5]. The existence of progesterone receptors on the mitochondrial membrane can also determine the influence of progestins on mitochondrial functions including the transcription of mitochondrial genes and metabolic processes [5].

**Figure 2:**
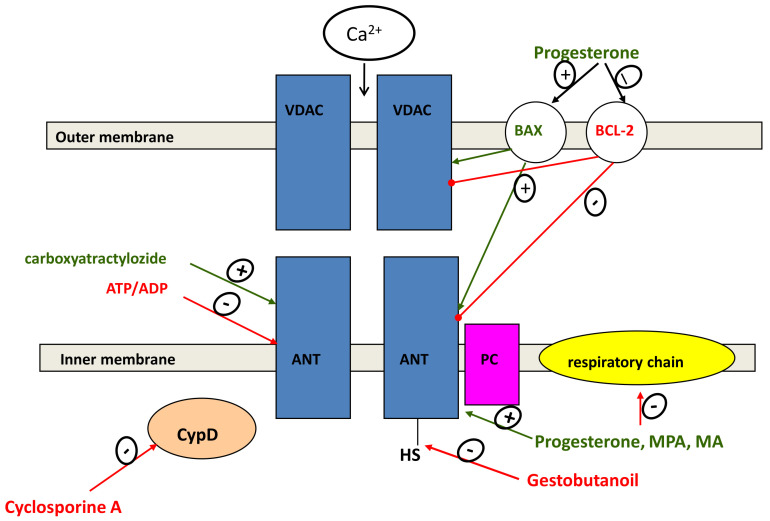
Influence of progesterone and progestins on MPTP opening. Progesterone and progestins decrease the expression ofBcl-2 and increase the expression of Bax, promoting MPTP opening. Cyclosporine A and ADP/ATP prevent MPTP opening via interaction with cyclophillin D and ANT, respectively. Progesterone, MPA and MA activate MPTP opening, and GB inhibits MPTP opening induced by calcium ions and pro-oxidants by decreasing or increasing their threshold concentration. The influence of GB is prevented by CAT, which indicates the participation of ANT in this effect. VDAC, ANT, and PC are the components of MPTP; OMM and IMM are the outer and the inner mitochondrial membranes. The resulting effect may depend on the concentration of the steroid in the region of the plasma membrane and mitochondria in tumor and non-transformed cells. The inhibition of the MPTP opening can decrease the cardiotoxicity of a number of anticancer agents, since the inhibitors of mitochondrial membrane permeability are known to have cardioprotective effects [54]. This property may be also useful in the treatment of neurodegenerative and ischemic diseases. Green line-activation, red line-inhibition of indicated processes.

As was shown, progestins can act on mitochondrial functions in a different manner depending on their chemical structure and concentration [54]. According to our data, progesterone, MPA and MA activated the MPTP opening and the mitochondrial swelling induced by calcium ions or reactive oxygen species, acting at different threshold concentrations [54]. As a rule, the activators of MPTP opening are cytotoxic drugs, and the inhibitors of MPTP opening are chemoprotective and chemosensitizing drugs. Thus, activators are doxorubicin, progesterone, pro-oxidants, tapsigargin, etc., and inhibitors are cyclosporin A, N-acetylcysteine, and antioxidants [54]. Presumably, different concentrations of progestin can modulate cell viability, both toward apoptosis and cytoprotection. This proposal was confirmed by experiments in which a combination of anticancer drugs (doxorubicin, vinblastine) with pregnane steroids decreased their cardiocytotoxic and hepatotoxic effects and increased the cytotoxic effect towards drug-resistant cancer cells [54]. Earlier observed a unique influence of pregnane progestogen, gestobutanoil, on the cyclosporine A-sensitive calcium-dependent MPTP opening, a key event in the initiation of apoptosis. GB has been shown to act as the thiol-binding reagent N-ethylmaleimide (NEM). At low concentrations and short-term exposure, GB, like NEM, inhibited MPTP opening, while at high concentrations and prolonged exposure it activated, like NEM, the production of reactive oxygen species [54]. Moreover, GB enhanced the cytotoxic effect of doxorubicin on cancer cells [54]. Thus, GB can either increase or decrease the cytostatic activity of chemotherapeutic drugs by inhibiting their transport from the cell or by inhibiting the MPTP opening, respectively (Figure 2).

It is possible that GB forms hydrogen bonds with the SH-groups of ANT or cyclophilin D. GB acts on tumor cells and mitochondria like cyclosporine A, an inhibitor of cyclophilin D and P-glycoprotein. It is known that both the knockout of the cyclophilin D gene and the application of cyclophilin D inhibitors exhibit high efficiency in a cardioprotection during preclinical trials on animals [55]. The participation and the role of pregnane steroids in the modulation of MPTP and MDR in cancer cells are of undoubted interest and need further research.

In this context, studies of the role of ANT in the resistance of tumor cells to apoptosis may be of great importance. As follows from our studies, derivatives of 17-acetate mepregenol containing a modified hydroxyl group in the 3-position of the pregnane framework inhibit MPTP opening while carboxyatractyloside, a selective ANT inhibitor, removes this effect [54]. It is known that overexpression or knockdown of ANT isoforms modulates sensitivity of cells to apoptotic stimuli in which different isoforms have opposite effects on cell survival [56,57]. Pro-apoptotic isoform ANT1 was found to be low in many cancers, while the induction of overexpression of ANT1 in breast cancer promoted the tumor apoptosis [58].

ANT1 and ANT3 isoforms act as pro-apoptotic factors while ANT2 and 4 isoforms provide the resistance to death inducing stimuli [59,60,61]. Thus, differences in the expression of certain ANT isoforms can also determine the resistance of cancer cells to apoptosis.

## 3. Progestins as Chemosensitizers, Possible Targets

MDR is also an actual problem with regard to anticancer therapy. The most common mechanism of MDR is the overexpression of transporters of the ATP binding cassette (ABC) family. These pumps reduce the intracellular accumulation of many anticancer drugs to lower their effective concentrations, thus decreasing or abolishing the chemotherapy efficacy.

Among seven human ABC subfamilies, namely ABCA-ABCG, ABCC is the largest subfamily, with 13 members. Nine of them are termed “multidrug resistance proteins” (MRPs1-9) due to their ability to mediate cancer MDR by extruding various chemotherapeutic agents from tumor cells [62]. Another MDR protein is P-gp (P-gp/ABCB1), a glycosylated 170-kDa transmembrane protein encoded by the MDR1 gene; is the best studied drug efflux pump of the family of ABC transporters [63]. Four generations of P-gp inhibitors have already been developed. One of them, tariqvidar, has been approved by the FDA. Tariquidar is planned to be introduced into clinical practice for the treatment of multidrug resistance in non-small cell lung cancer as a first-line therapy [64].

The first mention of progesterone as an MDR regulator was published in 1989 by Yang [65]. Many studies have been devoted to progesterone and its analogs as modulators of P-gp mediated resistance of tumor cells [66,67,68,69]. Moreover, to date, there is convincing evidence of the specific action of progesterone on P-glycoprotein [70].

Most P-gp inhibitors bind with the transmembrane domain of the transporter [71]. Steroids can probably inhibit the transporter by interacting with the nucleotide-binding domain of Pgp [62]. The model of the Pgp transport activity assumes the “pumping out” of the cytostatic agent from the cell due to the activation of ATP hydrolysis. But in a study on the highly resistant DC-3F/ADX line, the steroid hormones progesterone and deoxycorticosterone stimulated the ATPase activity of P-gp with activation constants of 20–25 µM and 40–50 µM, respectively, whereby the degree of resistance of the cell line decreased [72]. A similar result was obtained earlier in a study on an Sf9 cell culture, in which tamoxifen, progesterone, estradiol, hydrocortisone, and corticosterone stimulated ATPase of Pgp at a concentration of about 50 µM [73].

It can be assumed that steroids decrease drug resistance by directly affecting P-gp through the stimulation of ATPase in the direction of “pumping” of drug substances into the cell. This hypothesis was supported by the data on the effect of cholesterol, a precursor of steroid hormones, on the transport activity of P-gp. Cholesterol inhibited the transport of daunorubicin from human liver tumor cells NIH-G185, but at the same time, stimulated ATP hydrolysis. In this case, ATPase activity of P-gp increased, but the efflux of drugs was inhibited [74]. It is probable that the hydrophobicity and amphiphilicity are also important properties of steroids because of their ability to inhibit the transport activity of P-gp. Barnes et al., 1996, ref. [66], tested 26 steroid compounds to determine whether they can be transportedby P-gp and increase the accumulation of vinblastin in the cells of human colon carcinoma SW620 with 300-fold resistance to doxorubicin. It was shown that, among the steroids tested (cortisol, dexamethasone, corticosterone, aldosterone, cholesterol, estradiol, pregnenolone and progesterone), only progesterone was not transported by P-gp and at the same time inhibited the transport of these steroids. Moreover, progesterone increased the accumulation of vinblastine 21 times, medroxyprogesterone, which contains a more hydrophilic 17beta-hydroxy group, only six times, and medroxyprogesterone 17beta-acetate containing a hydrophobic side chain 21.3 times. MA was not tested in this study, but other experiments revealed that treatment of breast cancer cells with MA abolished the resistance to doxorubicin and vincristine [75]. Also, some other studies showed the reversing activity of MA towards resistant cells (Table 3). Until now, MA is predominantly used as a palliative cancer therapy and as a means of improving the quality and duration of life, including appetite recovery [5]. Different progestins or their combinations with other MDR modulators that reduce MDR are listed in Table 3.

Some well-known MDR modulators, specifically verapamil and bromocriptine, (Kd = 7×10^−9^ M) decrease the inhibition constant for progesterone, which makes it possible to use a lower concentration of the hormone in the treatment of tumors [91]. These data suggest that progestogens inhibit P-gp by binding to the specific site and blocking the efflux of cytostatics [95,96].

Progesterone also reduces Pgp expression [78] and decreases BCRP-mediated MDR in breast cancer cells, inhibiting the transcription via binding to the progesterone promoter in the gene encoding BCRP [75].

Our data obtained with the progestins GB and MPA also demonstrated the inhibition of P-gp activity in resistant cancer cell cultures Hep-2/R and MCF-7/R [97]. In the concentration of 10^−5^ M these progestins decreased P-gp activity to a greater extent than the reference drugs verapamil and cyclosporine A.

The expression of ABCG2/BCRP proteins is also considered as a reason for MDR [98]. Furthermore, the resistance to apoptosis, connected with the overexpression of Bcl-2 mitochondrial proteins, is an important part of the common MDR phenotype. Currently, Bcl-2 blockers are considered as modern promising drugs to overcome drug resistance. Venetoclax, a Bcl-2 inhibitor, is already available to treat chronic lymphocytic leukemia in patients with specific chromosomal abnormalities [99]. There is strong evidence that progesterone regulates bcl-2 expression in cancer and non-cancer cells. Progesterone via PR interacts with the bcl-2 promoter to induce its expression in leiomyoma tissue, which may explain, in part, the progesterone-dependent enhancement of growth in uterine leiomyoma [100].

In the case of breast cancer cells, Bcl-2 is down-regulated by progesterone, which has been confirmed in numerous studies. The up-regulation of Bclx-L and Bclx-S and a down-regulation of Bcl-2 mRNAs, which are specific to the MPA response and unrelated to apoptotic processes, were observed in T47-D, MCF-7 and H466-B cell lines [101]. As was shown, progesterone-induced Bcl-2 down-regulation is specific for the above mentioned PR-positive cells, but was not observed in the PR-negative MDA-MB-231 cells [101].

Bcl-2 down-regulation is not always connected only with apoptosis. Bcl-2 proteins prevent apoptosis, but also can prevent proliferation [102]. Consequently, the overexpression of Bcl-2 is not usually connected with metastasizing and poor prognosis. As was shown in a study on women with breast cancer, only one of 17 cases demonstrated elevated Bcl-2 in metastases [102].

Synthetic progestins, such as MPA, can act on Bcl-2 expression not only in breast cancer cells or endometrial cancer cells, but also in colonic carcinoma and pancreatic carcinoma cells, causing apoptosis by mechanisms that include Bcl-2downregulation and phosphorylation, which decrease Bcl-2 function [103]. P_4_ also suppresses colonic carcinoma by Bcl-2 down-regulation and caspase-3 up-regulation, as was shown in SW620 cells. It is important that the size of induced apoptosis was concentration-dependent, and a maximum effect was achieved at very high concentration of progesterone [104].

In the last decades, the membrane progesterone receptors (mPRα) and progesterone receptor membrane component 1 (PRGMC1) have been considered as possible participants in MDR. It has been recently shown that mPRα activated by progestin nomegestrole acetate (NMGA) caused Pgp mRNA up-regulation in HepG2 and Huh7 cells [105]. This was the first mention of mPR as a regulator of the drug efflux linked to MDR. The authors showed that NMGA activates the drug efflux by the induction of ATP synthesis. Also, NMGA could regulate the expression of MDR-related genes in a time-dependent manner [76]. The regulation was biphasic: 50 nM NMGA induced a rapid (15 min) down-regulation of the mRNA and protein expression level of MDR1 followed by the up-regulation of MDR1 expression during the long-term (five day) application. If a similar modulation of the MDR phenotype occurs in vivo, then the duration of treatment with NMGA becomes a very important factor to verify the time course of MDR modulation by reversal agents in clinical trials [76].

The novel effect of progesterone on the nucleoside transport was recently described, which can explain the biphasic regulation of Pgp by changes in ATPase activity. The effects of various steroids on nucleoside uptake were tested in NBTI, S-[4-(nitrobenzyl]-6-thioinosine)-sensitive cells, SH-SY5Y, and NBTI-insensitive H9c2 rat cardiomyoblasts. It was found that E2 and progesterone, at micromolar concentrations, markedly inhibited both NBTI-sensitive and insensitive uptake of thymidine [106]. The authors proposed that several non-specific and not well-studied effects of steroids, such as the immunosuppressive and antitumor effects of their high doses, may be mediated by the inhibition of nucleotide transport. Therefore, E2 and P4 may represent a new group of inhibitors of nucleotide transporters.

## 4. New MDR-Linked Targets of Progestin Action

New MDR-linked targets of the action of progestins are presumably connected with the progesterone receptor membrane component 1 (PRGMC1) functions [107]. PRGMC1 seems to be tissue-dependent. It can promote the cytoprotection in the brain and the heart after injury [108]. However, it is unclear whether PGRMC1 directly regulates neuroprotection. As was shown on cultured neurons, the neurotrophic activity of the PGRMC1 is realized with the involvement of MAP kinase and Akt signaling [108,109]. In cancer, PRGMC1 can protect cells from the action of chemotherapy drugs. PGRMC1 knockdown is associated with increased chemosensitivity of human endometrial xenograft tumors to doxorubicin, paclitaxel and carboplatin, followed by a fourfold decrease in the tumor volume in comparison with PGRMC1-intact controls [110]. Similar events occur in breast cancer cells, especially in PR-deficient ones. In triple-negative MDA-MB-231breast cancer cells, which express PGRMC1 but lack expression of the classical P_4_ receptor, PGRMC1 decreased the apoptotic effects of doxorubicin [111]. Since PRGMC1 modulates the activity of cytP450, its stimulation may be accompanied by an accelerated conversion of doxorubicin, thereby reducing its effect. The effect of other cytostatics metabolized by P450, in particular cisplatin, was also reduced [112].

If PRGMC1 is overexpressed, proliferative equilibrium shifts towards mitosis [113]. Recently it was shown that PGRMC1 interacts with EGFR, leading to the activation of the Wnt/β-catenin and NF-κB pathways, and promotes proliferation and resistance to anticancer drugs-protein kinase inhibitors such as erlotinib, for example [114].

PRGMC1 has been proposed to act as a progesterone signaling intermediate in multiple cell types [115]. Consequently, PRGMC1can modulate the effect of P_4_ and its synthetic analogues on cancer and non-cancer cells.

## 5. Role of Wnt-Signaling in MDR and Cell Proliferation

As was noted above, P_4_ and its synthetic analogue MPA inhibit Wnt signaling and reduce MDR in hormone-dependent cancers. Besides, both steroids down-regulate the expression of genes involved in TGF-β and Wnt/β-catenin signaling [24]. Ovarian hormones also affect Wnt-signaling in ovarian cancer cells. As was shown, treatment with oestradiol increased the level of β-catenin protein, whereas co-treatment with MPA provided an opposite effect [116].

In the Wnt/β-catenin signaling pathway, nuclear β-catenin can move the cyclic AMP response-element binding protein (CBP) into the promoter region of the MDR1 gene [41]. MDR1 is one of the target genes of TCF/LEF and a direct target of the TCF4/β-catenin transcription complex. The depletion of endogenous β-catenin can significantly reduce the transcription and expression of the MDR1 gene, which leads to the restoration of susceptibility to drug-induced apoptosis. Indeed, the sensitivity of colorectal cancer cells to oxaliplatin was increased with the inhibition of Wnt/β-catenin signaling [117]. Thus, in colorectal cancer, the Wnt/β-catenin signaling cascade contributes to the increased resistance of various chemotherapeutic agents through the activation of MDR1 [41].

Since one of the factors in the development of MDR is Wnt/β-catenin signaling pathway activation [118], the inhibitory effect of progesterone on this signaling pathway may also be a promising aspect in the clinical use of progestins as chemosensitizers.

Taken together, these data indicate that progesterone and synthetic progestins, which inhibit Wnt/β-catenin signaling, can negatively regulate the MDR1 gene, thus promoting the sensitivity to chemotherapy.

It should be noted that the same targets, characteristic of the action of progestins, are used in actual antitumor immunotherapy. This approach is applied to tumors that are resistant to conventional treatments. One of the targets actively studied in this direction, is TGF-β. As mentioned above, TGF-β signaling drives cancer progression and metastasis as well as the epithelial-to-mesenchymal transition. The TGF-β pathway has been pharmacologically targeted using TGF-β -directed monoclonal antibodies, ligand traps, antisense oligonucleotides, and vaccines [119,120,121]. Since this treatment has been successful only in some cases, it was concluded that TGF-β inhibition by single agents does not lead to direct cytotoxic activity and tumor regression. It is assumed that for more meaningful efficacy, combined approaches should be considered, and the choice of the right therapeutic partner is a fundamental aspect of this [119]. The same applies to Wnt/catenin signaling which promotes invasion, metastasis and therapeutic resistance. Wnt signaling-targeted monoclonal antibody and β-catenin inhibitors are undergoing clinical trials or preclinical studies for the treatment of Wnt-driven cancers. It is assumed that Wnt signaling-targeted therapeutic agents may also be useful in combination therapy with immune checkpoint blockers [122]. Taking into account corresponding aspects of our review, it can be assumed that progestins may be effective partners in antitumor immunotherapy, which requires experimental verification.

## 6. Conclusions

According to current data, progesterone and synthetic progestins participate in the regulation of cell proliferation, cell signaling and drug resistance. Unlike progesterone, which acts predominantly through its receptors and realizes typical progestin activity, synthetic progestins exhibit a variety of additional properties depending on their structure, concentration, and time of exposure. Some progestins have anticancer activity, which is associated with their cytotoxic and chemosensitizing action on different cancer cells. The detection of new targets of progestins, which include non-specific transporters of MDR and MPTP, actualizes their investigation and application, particularly in the case of hormone-dependent tumor cells overexpressing MDR-related proteins. The phenomenon of drug resistance reveals itself even towards newly synthesized drugs and significantly limits the efficacy of anticancer therapy.

It is assumed that the favorable influence of progestins is predominantly associated with their own cytotoxic effects on some hormone-dependent tumors (breast cancer, endometrial cancer, cervical cancer); the modulation of expression and activity of MDR-related proteins; the inhibition of survival signaling pathways, especially TGF-β and Wnt/β-catenin pathways which activate the proliferation and promote MDR in cancer cells; and the triggering of mitochondrial-dependent apoptosis. The inhibitory effect of progestins on these signaling pathways may be a promising aspect in their clinical use. By these ways, progestins can restore the proliferative balance, the ability for apoptosis, and the chemosensitivity to drugs. Further research is needed to determine their dosage and the mode of therapy, whether it be pulse or continuous, for the achievement of a targeted effect.

## Figures and Tables

**Table 1:** Progesterone and pregnane progestins with the anticancer and chemosensitizing activities.

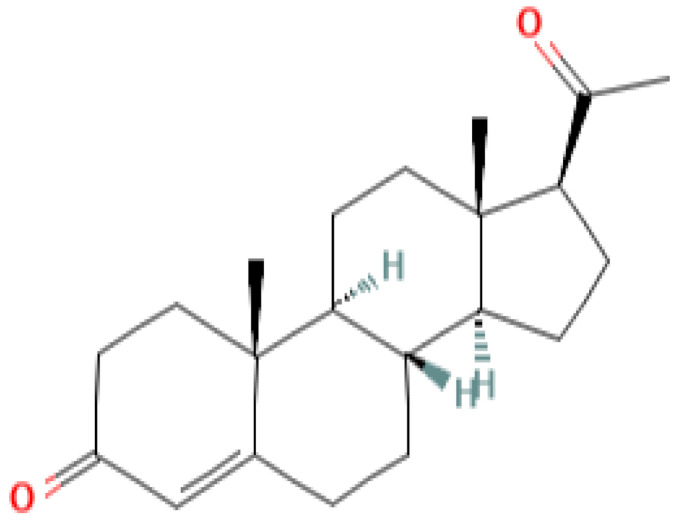	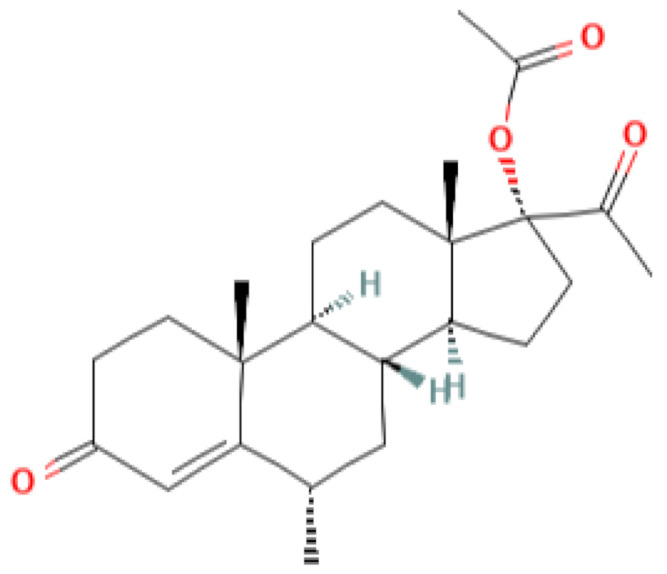	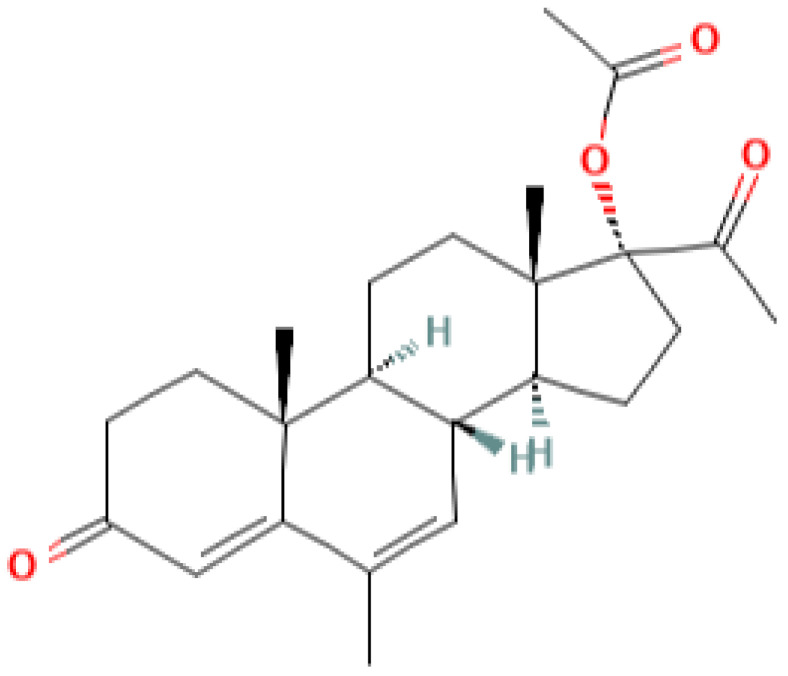	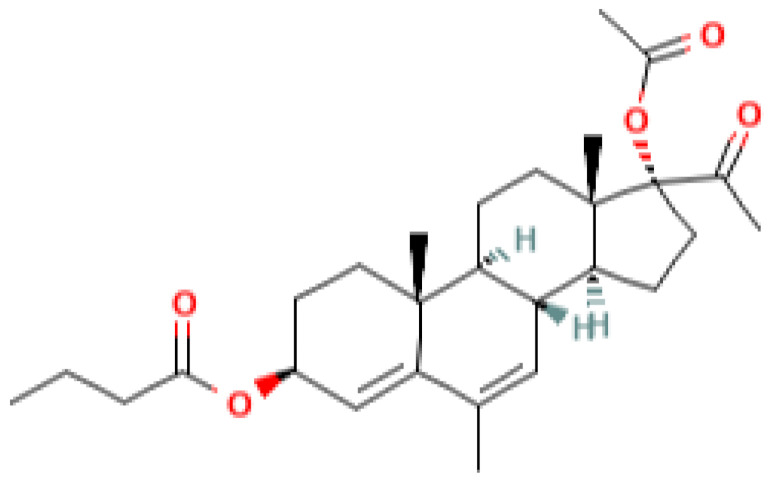
progesterone	medroxyprogesterone acetate (MPA)	megestrol acetate (MA)	gestobutanoyl (butagest, buterol) (GB)

**Table 2:** Completed and ongoing clinical trials of progestogens in endometrial hyperplasia, endometrial cancer, and breast cancer.

Type of Disease	Drugs	Estimated Performance Indicators	Clinical Trial Number
Endometrial hyperplasia	- MPA vs. Dydrogesterone; - Vaginal Micronized - Progesterone vs. LNG-IUS; - Megestrol acetate plus LNG-IUS; - LNG-IUS	- Complete response rate (CR) -Regression and remission rate of endometrial hyperplasia - Changes in the level of hormone receptor expression measured by immunohistochemistry; - Genes related to proliferation, estrogen signaling, and Wnt signaling	NCT03675139 NCT03992937 NCT03241888 NCT00788671
Endometrial Cancer	- PD-1 inhibitor combined with progesterone; - MPA vs. MPA plus Entinostat; - LNG; - MPA; - LNG-IUS with or without everolimus; - MA with or without Temsirolimus and Tamoxifen; -MPA + Tamoxifen vs. Everolimus + Letrozole	- Expression of the PR; co-expression of ER; Ki67 and p21; - Hyperplastic or neoplastic lesion; - Fertility rate; - Frequency of the endometrial adenocarcinomas of the uterine corpus; - Life expectancy, number of relapses, side effects	NCT04046185 NCT03018249 NCT03463252 NCT00064025 NCT02397083 NCT03077698 NCT00729586 NCT02228681
Cervical Intraepithelial Neoplasia	Progesterone	Regression rates of CIN I and II	NCT00247169
Breast Cancer	- Megestrol Acetate + letrolzole vs. letrozole; - Exemestane vs. - Megestrol Acetate; - Megestrolacetate; - Megestrol Acetate vs. MPA; - MPA with or without Cyclophosphamide & Methotrexate; - Depot hydroxy-progesterone; - Letrozole vs. Letrozole plus Prometrium vs. Tamoxifen + Prometrium	- Determination of change in tumor proliferation; Geometric mean suppression of proliferation marker Ki67; - Definition of a gene set as a predictive biomarker for a reduction in Ki67; - Changes in the apoptotic markers Bcl-2 and Caspase 3 in the tumors following intervention; - Changes in ER, PR, AR, FoxA1, Cyclin D1 protein and mRNA expression in the tumors following intervention	NCT03306472 NCT01237327 NCT00005975 NCT03024580 NCT00002920 NCT00577122 NCT00123669 NCT03906669

**Table 3:** Progestins modulating MDR in experimental models.

Cell Line/Object	Progestin or Combination Progestin + Modulator	Cytostatic Drug	Progestin Effect	Ref.
K562	megestrole + droloxifene	adriamycin	significantly inhibit mdr1 and GST pi expression	[76]
In vivo, patients	high-dose oral megestrol acetate	vinblastine	in vivo decrease the general toxicity of vinblastine	[77]
Breast carcinoma MCF-7/ADR	nomegestrol acetate	adriamycin	Downregulating the mRNA and protein expression levels of MDR1 and GSTpi, increasing intracellular drug concentration and arresting cells at the G2M phase (NOM in combination with ADR)	[78]
Colon carcinoma HCT-116/VM46, breast carcinoma MCF-7/ADR, murine cell line, J774.2	megestrol acetate	doxorubicin	increasing DOX accumulation measured by flow cytometry method	[79]
Human neuroblastic SH-SY5Y/VCR cells	megestrol acetate	vincristine	steroids inhibited the binding to P-glycoprotein	[80]
Human ovarian cancer cell lines, ES-2, TOV-21G, and UWB1.289; Endometrial cancer cell lines, HEC-1A and HEC-59	progesterone-calcitriol	cisplatin	increased caspase-3, BAX, and decreased bcl-2, progesterone-calcitriol potentiated the anti-growth effects of cisplatin on cancer cells by attenuating the expression of SMAD2/3, multidrug resistance protein- 1 (MDR-1), and ABC transporters (ABCG1, and ABCG2),	[81]
Ovarian cancer cells, ES-2, and TOV-21, OV-90, TOV-112D, HEC-1A, and HEC-59	progesterone-calcitriol	-	progesterone induced apoptosis through activation of caspase-8	[82]
K562/R7 erythroleukaemiacells, steroidogenic NCI-H295R adrenocortical carcinoma cells	pregnane steroids	doxorubicine	interactions of pregnane modulators with human progesterone and pregnane X receptors	[70]
HL60	pregnane steroids	mitoxantrone	specific P-gp, MRP1 and BCRPbinding and blocking	[70]
MCF7/MTX	pregnanesteroids	mitoxantrone	specific P-gp, MRP1 and BCRPbinding and blocking	[70]
P-gp-positive human colon cancer cell lines (HT-29 and CACO-2) and the human T-cell leukemia cell line (HUT-102)	megestrol acetate	vincristine	augmentation of vincristine cytotoxicity	[83]
Mice, Female	progesterone		influence MDR-1 transcript levels	[84]
P-gp-positive Chinese hamster ovary cells (CHO)C5 cells	progesterone	deoxycorticosterone	P-gp, mitochondrial electron transport chain interaction	[85]
T47D, progesterone receptor positive (PR+)	progesterone	mitoxantrone	Progesterone negatively regulates BCRP	[75]
PR-negative MDA-MB-231 cells	progesterone	mitoxantrone	Progesterone negatively regulates BCRP	[75]
Human colon cancer line HCT-15	progesterone-derived carbamates	paclitaxel and doxorubicin	increase in intracellular accumulation and reduced drug efflux	[86]
Human placental BeWo cells	progesterone	-	progesterone up-regulates BCRP via a mechanism other than PR, presumably via a nonclassical PR	[87]
MCF-7/ADR (adriamycin resistant)	medroxyprogesterone acetate + cyclosporine	adriamycin	enhancement of adriamycin cytotoxicity	[88]
CHO/ADR	progesterone + bromocriptine	vincristine	non-competitively inhibits the vinblastine-dependent P-gp ATPase activity	[89]
Chinese hamster DC-3F lung fibroblasts and its DC3F/ADX resistance variant	progesterone + verapamil	vincristine	progesterone and verapamil induce a synergistic modulation of P-gp ATPase activity	[90]
CH^R^B30	progesterone + verapamil	adriamycin	inhibition of ATPase activity	[91]
U87MG Human Glioblastoma Cells	Progesterone alone and in combination with TMZ	TMZ	progesterone suppressed EGFR/PI3K/Akt/mTOR signaling pathway and MGMT expression; enhanced the cytotoxic effects of TMZ and reduced its toxic side effects in healthy primary cells.	[92]
MCF-7/dox	gestobutanoyl	etoposide, doxorubicin	MRP1, BCRP, BCL-2 mRNA expression inhibition	[93]
Caco-2	progesterone	-	inhibition P-gp activity (100µM)	[94]

## Data Availability

Not applicable.

## Abbreviations

ADR	adryamicin (doxorubicine)
ACC	adrenocortical carcinoma
ANT	adenylate translocase
BCRP	breast cancer resistance protein
CAT	carboxyatractyloside
CHOP	CCAAT/enhancer-binding protein homologous protein
CRC	colorectal cancer
EC	endometrial cancer
EMT	epithelial-to-mesenchymal transition
ER	estrogen receptor
ERK-1 and ERK-2	extracellular signal-regulated kinase (ERK)1/2
FDA	Food and Drug Administration
FOXO1	forkhead box O1A
GB	pregnan progestogen, gestobutanoil
GPR30	G protein-coupled receptor 30
HER2	human epidermal growth factor receptor-2
HERPUD1	homocysteine-responsive endoplasmic reticulum-resident ubiquitin-like domain member 1 protein
LBD	ligand binding domain
MA	megestrol acetate
MAP	kinase-mitogen-activated protein kinase
MDR	multidrug resistance
MGMT	O6-methylguanine-DNA-methyltransferase
MPA	medroxyprogesterone acetate
mPRα	membrane progesterone receptor alpha
MPTP	mitochondrial permeability transition pore
MMP9	Matrix metallopeptidase 9
MRP	multidrug resistance proteins
NBD	nucleotide binding domain
NBTI	S-[4-nitrobenzyl]-6-thioinosine
NEAT1	NEAT1 Nuclear enriched abundant Transcript-1
NEM	N-ethylmaleimide
NMGA	nomegestrol acetate
nPR	nuclear progesterone receptor
OSE	ovarian surface epithelium
P4	progesterone
P-gp	P-glycoprotein
PR	progesterone receptor
PRGMC1	progesterone receptor membrane component 1
SERM	Selective estrogen receptor modulator
TGF-β	transforming growth factor β
TMD	transmembrane domain
TMZ	Temozolomide
TIL	tumor-infiltrating lymphocytes
TSPO	translocator protein or mitochondrial benzodiazepine receptor
